# Chemical Exposures: No Dental Dilemma for BPA

**Published:** 2006-07

**Authors:** Julian Josephson

Among the many uses of bisphenol A (BPA) is the manufacture of resin-based dental composites and sealants. Recently a team of researchers from the CDC sank their teeth into questions about whether BPA monomer leaching from sealants could be harmful to people. The results of their human study, presented in the March 2006 issue of the *Journal of the American Dental Association*, suggest that although leaching does occur, sealants are still a safe means of preventing dental cavities.

Low-level exposures to BPA monomer in pregnant rodents, at a level that humans could potentially receive from dental sealants, have been shown to disrupt reproductive development in their fetuses, and concerns have emerged about the possibility of human health effects from dental exposures. Scientific exploration of this question has yielded inconsistent results, says Renée Joskow, first author of the March paper. Much of this is due to limitations in laboratory detection and translation of animal studies to human health effects, as well as insufficiently addressing the parameters of exposure in a clinical dental setting.

The CDC team, led by Joskow (now of the U.S. Public Health Service) and Dana Barr, looked at 14 nonsmokers receiving their first resin-based sealants as part of their routine dental care. Each subject received one of two brands of dental sealant manufactured by two well-established dental equipment and material supply firms. Then their saliva and urine were tested for BPA.

All the patients had BPA in their saliva and urine, even before treatment. For patients receiving Helioseal F sealants, saliva BPA doubled immediately after treatment and returned to baseline within 1 hour. Urine BPA more than tripled 1 hour after treatment and returned to baseline within 24 hours. For patients receiving Delton LC sealants, saliva BPA increased nearly 126 times immediately after application and was still 23 times higher after 1 hour. Urine BPA jumped 10 times 1 hour after treatment and was still elevated 24 hours later. Both levels eventually returned to baseline.

Barr believes the patients’ baseline BPA came from background exposures from environmental sources such as water and food packaging. These, she suggests, could be “a more chronic low-level source of exposure” than dental sealants. Barr adds that in her view, although point-source exposure from dental sealants might approach levels that induce health effects in rodents, “[it] is not the most significant source of exposure in humans.” Moreover, she holds that exposure to BPA from dental sealants, already variable and short-lived in the body, could be easily reduced further by having the patient spit frequently in the hours after application.

## Figures and Tables

**Figure:**
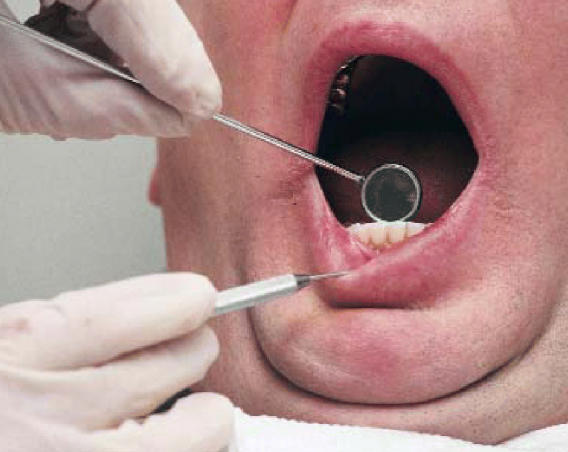
BPAhhh New data show that exposure to bisphenol A in dental sealants is likely an insignificant source of risk.

